# The Association of American Medical Editors

**Published:** 1886-06

**Authors:** 


THE ASSOCIATION OF AMERICAN MEDICAL EDITORS met in St. Louis on May 3rd. The annual address of the President, Professor H. O. Walker, M. D., of Detroit, was delivered and the annual election resulted in the selection of

Professor John V. Shoemaker, M. D., of Philadelphia, for president.

The Association was tendered a banquet by the Medical Press and Library Association of St. Louis.

